# Decreased plasma APOA1 levels are associated with increased severity of placenta accreta spectrum disorders: a nested case-control study

**DOI:** 10.3389/fendo.2025.1627377

**Published:** 2025-07-24

**Authors:** Shuai Zeng, Jiangxue Qu, Hai Jiang, Huifeng Shi, Jie Yan, Yangyu Zhao, Lian Chen

**Affiliations:** ^1^ Department of Obstetrics and Gynecology, Peking University Third Hospital, Beijing, China; ^2^ National Clinical Research Center for Obstetrics and Gynecology, Peking University Third Hospital, Beijing, China; ^3^ National Center for Healthcare Quality Management in Obstetrics, Peking University Third Hospital, Beijing, China; ^4^ State Key Laboratory of Female Fertility Promotion, Department of Obstetrics and Gynecology Peking University Third Hospital, Beijing, China

**Keywords:** apolipoprotein A1, plasma biomarker, blood transfusion, hysterectomy, invasive placenta accreta spectrum

## Abstract

**Objective:**

Placenta accreta spectrum (PAS) disorders are a series of gestational diseases, with severe adverse outcomes. Apolipoprotein A1 (APOA1) is a lipid molecule that plays a role in cell invasion, inflammation and immune response. This study aimed to elucidate the relationship between APOA1 and PAS, as well as its adverse outcomes.

**Methods:**

This is a nested case-control study involving 118 patients with PAS and 118 non-PAS control women. Plasma APOA1 levels were evaluated at gestational weeks 24^+0^ to 35^+6^ by enzyme-linked immunosorbent assay. The clinical characteristics and pregnancy outcomes were recorded and analyzed in relation to APOA1 levels.

**Results:**

The plasma APOA1 level in the PAS group was observed to be lower than that in the non-PAS group (*p* = 0.035). From 24^+0^ to 35^+6^ weeks of gestation, the trajectory of plasma APOA1 levels in the placenta percreta (PP) and placenta increta group exhibited a discernible decline. Maternal plasma APOA1 is a significant biomarker for the diagnosis of PAS and its adverse outcomes, particularly in the 32^+0^ to 35^+6^ weeks of gestation range for invasive PAS (AUC = 0.761, 95% CI 0.660-0.863, *p* < 0.001), PP (AUC = 0.889, 95% CI 0.801-0.976, *p* < 0.001), blood transfusion (AUC = 0.729, 95% CI 0.620-0.838, *p* < 0.001) and hysterectomy (AUC = 0.884, 95% CI 0.790-0.978, *p* < 0.001).

**Conclusions:**

A reduction in maternal plasma APOA1 levels was associated with the severity of PAS. APOA1 may serve as a biomarker for invasive PAS, blood transfusion and hysterectomy in late gestation.

## 1 Introduction

Placenta accreta spectrum (PAS) disorders are severe complications during pregnancy, characterized by an anomalous attachment of placenta villi to the uterine wall (1). In accordance with the classification system of the International Federation of Gynecology and Obstetrics (FIGO) (2), PAS can be subdivided into three categories: placenta accreta (PA), placenta increta (PI) and placenta percreta (PP). The prevalence of PAS has increased significantly over the past few decades, with an incidence of approximately 0.17% in 2019 (3, 4). The risk of accreta with placenta previa in women with a history of cesarean section (CS) is estimated to be 11% (5). Blood transfusion and even hysterectomy are typically inevitable in PAS patients, with an incidence of 46.9% and 52.2%, respectively (3). As the CS rate has increased globally (6, 7), the risk of PAS in women who have undergone a CS is high in the event of a subsequent pregnancy. It is important to recognize the potential risk associated with PAS and its adverse outcomes.

Ultrasound is a widely utilized diagnostic tool for distinguishing PAS during pregnancy. However, the reported prediction accuracies vary considerably among studies (8–10). Our group has published an ultrasound scoring system for PAS in 2018 (11), which has been extensively promoted in China. It is highly effective in predicting the presence of PAS; however, it is not so accurate to differentiate between PI and PP. Non-invasive biomarkers represent an alternative avenue for predicting PAS severity. Despite extensive research into potential proteins and small molecules (12), the predictive value of these biomarkers remains unsatisfactory and the sample size was relatively small. Furthermore, few studies have elucidated the correlation between biomarkers and adverse pregnancy outcomes of PAS.

Alterations in maternal plasma biomarkers reflect underlying pathological changes in placental biological function. While the precise pathogenesis of PAS remains incompletely understood, current evidence implicates two key mechanisms: defective decidualization and excessive trophoblast invasion (13). Apolipoprotein A1 (APOA1), the principal apolipoprotein of high-density lipoprotein (HDL), has been predominantly studied in gestational diabetes mellitus due to its canonical role in lipid metabolism (14, 15). However, emerging research has uncovered its involvement in other gestational disorders. A 2023 study demonstrated elevated peripheral APOA1 levels in preeclampsia patients and further established through *in vitro* experiments that APOA1 significantly inhibits trophoblast proliferation and invasive capacity (16). This finding suggests APOA1 may serve as a critical regulator of placental development. Interestingly, PAS and preeclampsia appear to represent opposite ends of the placentation: while PAS is characterized by excessive trophoblast invasion, preeclampsia features inadequate placental invasion (17, 18). This pathophysiological dichotomy led us to hypothesize that APOA1 levels might be differentially altered in PAS pregnancies. Furthermore, the tumor-like behavior of PAS trophoblasts - exhibiting both hyperinvasive properties and malignant proliferation patterns - provides additional rationale for investigating APOA1’s role (13). In oncology, APOA1 has been shown to regulate tumor proliferation, modulate the immune microenvironment and correlate with the prognosis (19, 20), suggesting potential parallel functions in placental pathophysiology. Based on these observations, we postulated that: APOA1 levels would vary significantly across PAS severity subtypes and correlate with clinical outcomes. Accordingly, the present study was undertaken to ascertain whether there is a correlation between plasma APOA1 levels and the likelihood of developing PAS, as well as the adverse outcomes.

## 2 Methods

### 2.1 Study design and data collection

**Figure 1:**
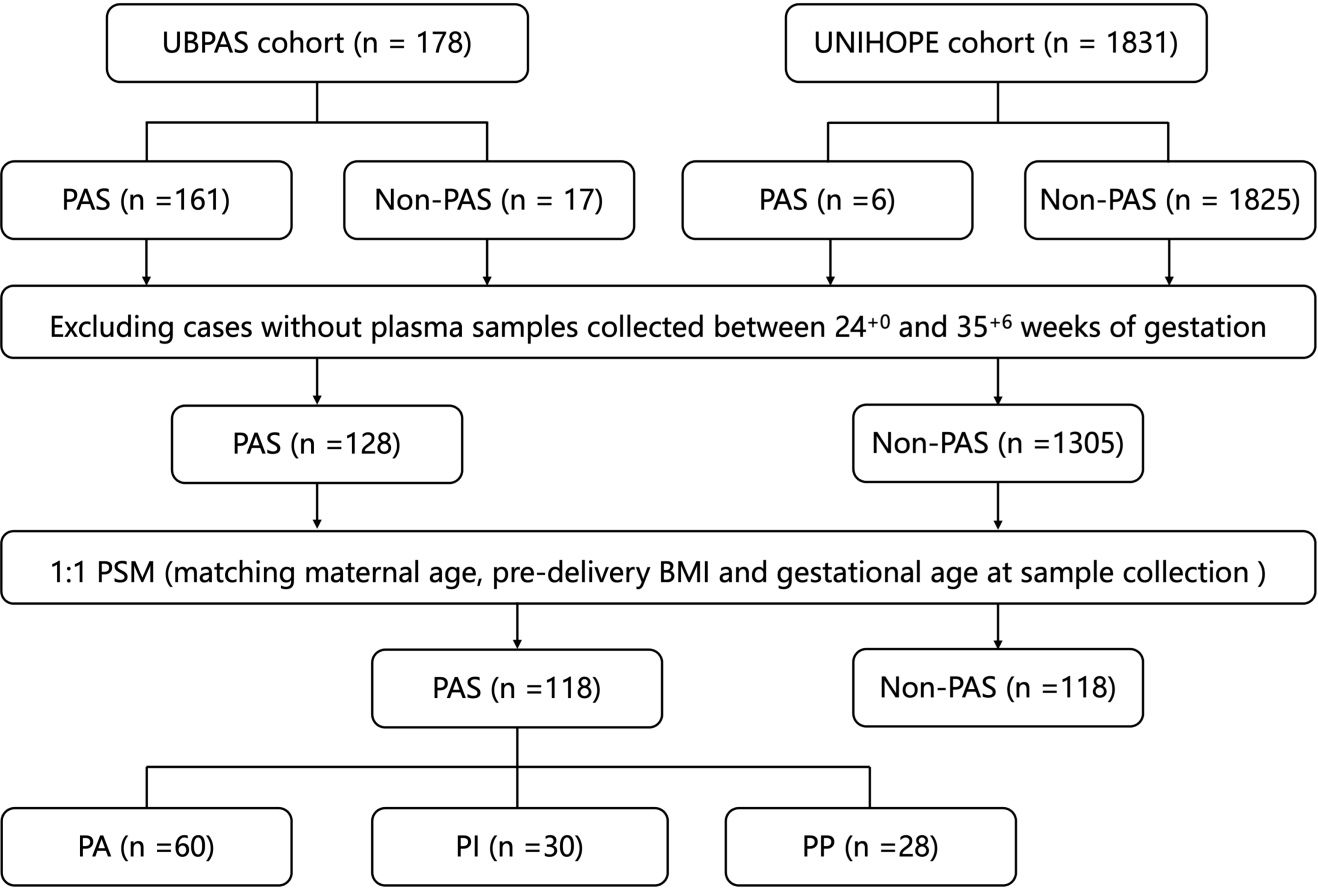
Flowchart of cases and samples selection. PAS, placenta accreta spectrum; PSM, propensity score matching; BMI, body mass index; PA, placenta accreta; PI, placenta increta; PP, placenta percreta.

A nested case-control study was conducted between 2019 and 2022 based on two cohorts: the Ultrasound Based PAS Screening (UBPAS) cohort and the University Hospital Advanced Age Pregnant (UNIHOPE) cohort in Peking University Third Hospital. As demonstrated in our previous study (11), the presence of PAS ultrasound scores of more than five has been shown to be associated with a high risk of invasive PAS and hysterectomy. Consequently, pregnant women who had a score of more than five would be recruited into the UBPAS cohort. All participants provided written informed consent. This study was conducted in accordance with the Declaration of Helsinki and received approval from the Peking University Third Hospital Medical Science Research Ethics Committee (IRB00006761-M2020262). PAS was diagnosed according to the 2019 FIGO classification system (2). After matching maternal age, pre-delivery body mass index (BMI) and gestational age at sample collection, controls were selected with the same sample size among pregnant women without PAS using the propensity score matching (PSM) method from the UNIHOPE cohort (**Figure 1**).

The clinical characteristics of the participants, including the maternal age, gravidity (except the current pregnancy), parity (except the current pregnancy), history of CS, history of curettage, pre-pregnant BMI, pre-delivery BMI, the presence of placenta previa, hypertension disorders of pregnancy, gestational diabetes mellitus, thyroid disease, gestational age at sample collection, gestational age at delivery, blood loss volume during vaginal delivery or CS, blood transfusion, hysterectomy, neonatal weight, and neonatal intensive care unit (NICU) admission were recorded. All surgeries were performed by our center’s experienced PAS team. The choice between uterine preservation and hysterectomy (placenta *in situ*) depended on preoperative imaging evaluation and intraoperative findings. Our “Nine-Step Uterine-Preservation Protocol” included: (1) uterine incision selection, (2) fetal delivery, (3) hemostasis (intrauterine tamponade or temporary uterine artery occlusion with tourniquet), (4) bladder dissection, (5) placental removal (dual-incision if needed), (6) uterine artery descending branch suture ligation, (7) cervical traction sutures, (8) closure of all uterine incisions, and (9) bladder integrity check (methylene blue). Hysterectomy was performed for severe PP, significant hemorrhage, unstable vital signs, or other life-threatening complications.

### 2.2 APOA1 quantification and statistical analysis

Peripheral blood samples were collected from participants between 24^+0^ and 35^+6^ weeks of gestation, following a minimum of eight hours of fasting. The samples were immediately placed on ice and then centrifuged at a force of 3000 rpm for 10 minutes at 4°C within two hours. The plasma was preserved at a temperature of -80°C. Plasma APOA1 levels were determined using enzyme linked immunosorbent assay (abx50705, Abbexa, the UK).

Student’s *t*-test, one-way ANOVA, Wilcoxon rank-sum test or Chi-square (χ^2^) tests were employed to conduct comparisons according to the type of variables being analyzed. A simple linear regression model illustrated the trajectory of APOA1 levels. To assess the relationship between APOA1 and PAS and associated outcomes, logistic and linear regression were employed. The area under receiver-operating-characteristics (ROC) curve (AUC) was utilized to demonstrate the sensitivity and specificity of APOA1 in predicting the occurrence of PAS and adverse outcomes. The statistical analyses and graphical representations were conducted using the following software: SPSS 21.0 (SPSS, Chicago, IL), R version 4.0.2 (R Core Team, Vienna, Austria) and GraphPad Prism 10.1.1 (GraphPad Software Inc., La Jolla, CA).

## 3 Results

### 3.1 Clinical characteristics of PAS patients and non-PAS women

A total of 118 patients with PAS (including 60 with PA, 30 with PI and 28 with PP) and 118 non-PAS women (control group) were included in the study. **Table 1** presents the clinical characteristics of the two groups.

**Table 1:** Clinical characteristics of the control and PAS groups.

Clinical characteristics	Control (n = 118)	PAS				*p* ^*^	*p* ^**^
		Total (n = 118)	Accreta (n = 60)	Increta (n = 30)	Percreta (n = 28)		
Age (years)	36.03 ± 3.32	35.58 ± 4.27	35.12 ± 4.63	36.30 ± 4.25	35.79 ± 3.42	0.368	0.419
Gravidity, n (%)							
0	25 (21.2)	12 (10.2)	9 (15.0)	3 (10.0)	0		
1	40 (33.9)	24 (20.3)	14 (23.3)	8 (26.7)	2 (7.1)		
≥2	53 (44.9)	82 (69.5)	37 (61.7)	19 (63.3)	26 (92.9)	0.001	<0.001
Parity, n (%)							
0	54 (45.8)	30 (25.4)	21 (35.0)	8 (26.7)	1 (3.6)		
1	62 (52.5)	61 (51.7)	32 (53.3)	15 (50.0)	14 (50.0)		
≥2	2 (1.7)	27 (22.9)	7 (11.7)	7 (23.3)	13 (46.4)	<0.001	<0.001
History of CS, n (%)							
No	88 (74.6)	40 (33.9)	29 (48.3)	8 (26.7)	3 (10.7)		
Yes	30 (25.4)	78 (66.1)	31 (51.7)	22 (73.3)	25 (89.3)	<0.001	<0.001
History of curettage, n (%)							
No	69 (58.5)	46 (39.0)	20 (33.3)	19 (63.3)	7 (25.0)		
Yes	49 (41.5)	72 (61.0)	40 (66.7)	11 (36.7)	21 (75.0)	0.003	<0.001
Pre-pregnancy BMI (kg/m^2^)	22.04 ± 2.93	22.59 ± 3.33	21.45 ± 2.53	23.60 ± 3.27^a,b^	23.93 ± 4.09^a,b^	0.181	<0.001
Pre-delivery BMI (kg/m^2^)	27.16 ± 3.25	27.42 ± 3.17	26.60 ± 2.34	28.18 ± 4.02^b^	28.37 ± 3.35^b^	0.528	0.037
Placenta previa, n (%)							
No	104 (88.1)	26 (22.0)	19 (31.7)	3 (10.0)	4 (14.3)		
Yes	14 (11.9)	92 (78.0)	41 (68.3)	27 (90.0)	24 (85.7)	<0.001	<0.001
Hypertension disorders of pregnancy, n (%)							
No	111 (94.1)	93 (78.8)	48 (80.0)	23 (76.7)	22 (78.6)		
Yes	7 (5.9)	25 (21.2)	12 (20.0)	7 (23.3)	6 (21.4)	0.001	0.003
Gestational diabetes mellitus, n (%)							
No	111 (94.1)	78 (66.1)	44 (73.3)	17 (56.7)	17 (60.7)		
Yes	7 (5.9)	40 (33.9)	16 (26.7)	13 (43.3)	11 (39.3)	<0.001	<0.001
Thyroid disease, n (%)							
No	99 (83.9)	99 (83.9)	48 (80.0)	23 (76.7)	28 (100.0)		
Yes	19 (16.1)	19 (16.1)	12 (20.0)	7 (23.3)	0	1.000	0.029
Gestational age at sample collection (weeks)	31.43 (27.96-34.29)	31.29 (27.96-34.14)	30.71 (26.32-33.39)	33.43 (29.64-35.14)	31.21 (28.07-32.82)	0.948	0.149
Gestational age at delivery (weeks)	39.14 (38.29-39.89)	35.71 (34.29-37.32)	37.14 (35.79-38.43)^a^	35.57 (35.25-36.50)^a^	33.86 (33.14-34.36)^a, b^	<0.001	<0.001
Mode of delivery, n (%)							
Vaginal	57 (48.3)	9 (7.6)	8 (13.3)	1 (3.3)	0		
CS	61 (51.7)	109 (92.4)	52 (86.7)	29 (96.7)	28 (100.0)	<0.001	<0.001
Blood loss volume (mL)	200.00 (200.00-300.00)	800.00 (500.00-1125.00)	500.00 (400.00-800.00)^a^	1000.00 (600.00-1200.00)^a,b^	1300.00 (825.00-2400.00)^a,b^	<0.001	<0.001
Blood transfusion, n (%)							
No	118 (100.0)	70 (59.3)	53 (88.3)	17 (56.7)	0		
Yes	0	48 (40.7)	7 (11.7)	13 (43.3)	28 (100.0)	<0.001	<0.001
Hysterectomy, n (%)							
No	118 (100.0)	97 (82.2)	60 (100.0)	29 (96.7)	8 (28.6)		
Yes	0	21 (17.8)	0	1 (3.3)	20 (71.4)	<0.001	<0.001
Neonatal weight (g)	3311.99 ± 479.53	2613.64 ± 600.85	2852.67 ± 540.52^a^	2543.33 ± 583.97^a,b^	2176.79 ± 478.89^a,b,c^	<0.001	<0.001
NICU admission, n (%)							
No	113 (95.8)	53 (44.9)	43 (71.7)	10 (33.3)	0		
Yes	5 (4.2)	65 (55.1)	17 (28.3)	20 (66.7)	28 (100.0)	<0.001	<0.001

PAS, placenta accreta spectrum disorders; CS, cesarean section; NICU, neonatal intensive care unit.

Age, pre-pregnancy BMI, pre-delivery BMI, and neonatal weight are shown as average ± standard deviation. Gestational age at sample collection, gestation age at delivery and blood loss volume are shown as median (*P*_25_-*P*_75_). Gravidity, parity, history of CS, history of curettage, placenta previa, hypertension disorder of pregnancy, gestational diabetes mellitus, thyroid disease, blood transfusion, hysterectomy and NICU admission are shown as number (percentage).

*p* ^*^, compared between the control and total PAS groups.

*p* ^**^, compared among the control group, accreta, increta and percreta groups.

a *p* < 0.05, compared with the control group.

b *p* < 0.05, compared with the placenta accreta groups.

c *p* < 0.05, compared with the placenta increta groups.

The PAS group exhibited higher prevalence of gravidity, parity, history of CS and curettage compared to the control group (*p* < 0.05). The proportions of placenta previa, hypertension disorders of pregnancy, gestational diabetes, hysterectomy, blood loss volume and NICU admission were higher in the PAS group than in the control group (*p* < 0.05). Conversely, the gestational age at delivery and neonatal weight were lower in the PAS group than in the control group (*p* < 0.05). No statistically significant differences were observed in other variables between the two groups (*p* > 0.05). When these clinical characteristics were compared among the control, PA, PI and PP subgroups, the differences aforementioned remained statistically significant (*p* < 0.05).

### 3.2 Maternal plasma APOA1 levels in the control and PAS groups

**Figure 2:**
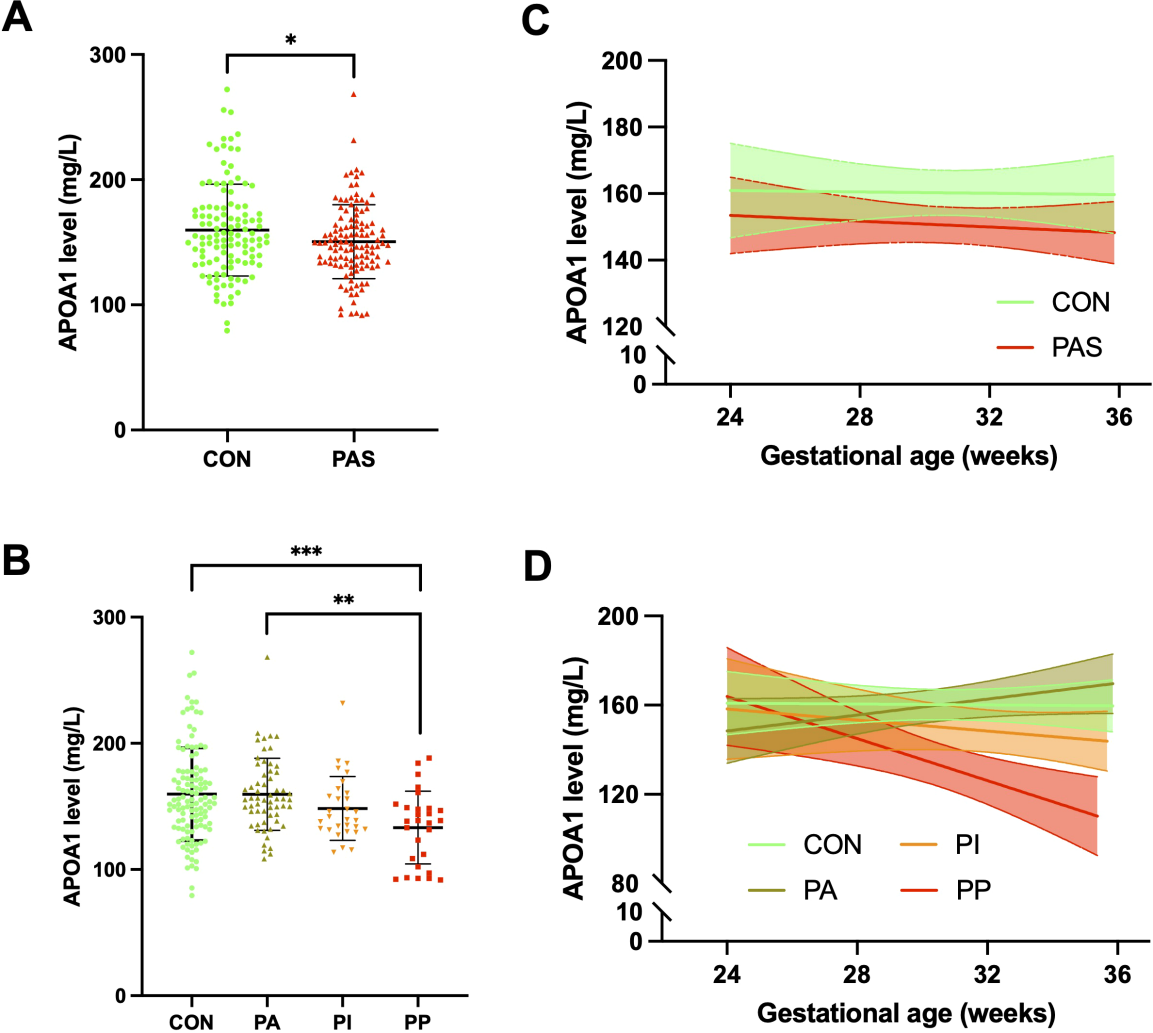
Plasma APOA1 levels in PAS patients and healthy pregnant women. **(A)** APOA1 levels in the control group and PAS group. **(B)** APOA1 levels in the control, PA, PI and PP group. **(C)** APOA1 trajectory in the control and PAS group between 24–36 weeks of gestation. **(D)** APOA1 trajectory in the control, PA, PI and PP group between 24–36 weeks of gestation. APOA1, apolipoprotein A1; PAS, placenta accreta spectrum; PA, placenta accreta; PI, placenta increta; PP, placenta percreta. *p < 0.05, **p<0.01, ***p<0.001.

A comparison of maternal plasma APOA1 levels revealed that PAS patients exhibited a significantly lower level (150.51 ± 29.58 mg/L) than control women (159.73 ± 36.74 mg/L) (*p* = 0.035) (**Figure 2A**). A comparison of the subgroups of PAS and the control group revealed that the PP patients exhibited a further reduction in APOA1 levels (133.24 ± 28.81 mg/L) compared to the PA patients (159.62 ± 28.50 mg/L) and the control group (*p* < 0.010). Nevertheless, the APOA1 levels of the PP group did not differ statistically from those of the PI group (148.41 ± 25.34 mg/L) (*p* = 0.291) (**Figure 2B**). Between 24^+0^ and 35^+6^ weeks of gestation, a slight decrease was observed in the trajectory of APOA1 levels in the PAS group, while no change was noted in the control group (**Figure 2C**). In subgroups analysis, it was evident that APOA1 trajectory in PP and PI group exhibited a notable decline (**Figure 2D**). As illustrated in **Table 2**, APOA1 levels were significantly lower in PP group than in the other groups in the 32^+0^-35^+6^ weeks gestational age range (*p* < 0.001).

**Table 2:** Plasma levels of APOA1 (mg/L) in different gestational ages in the control and PAS groups.

Gestational age	Control (n = 118)	PAS				*p* ^*^	*p* ^**^
		Total (n = 118)	Accreta (n = 60)	Increta (n = 30)	Percreta (n = 28)		
32^+0^-35^+6^ weeks	158.12 ± 37.45 (n = 50)	145.60 ± 29.19 (n = 51)	163.72 ± 19.97 (n = 23)	145.67 ± 20.19 (n = 16)	110.79 ± 22.59^a,b,c^ (n = 12)	0.064	< 0.001
28^+0^-31^+6^ weeks	162.79 ± 38.53 (n = 39)	158.59 ± 32.68 (n = 38)	166.94 ± 34.76 (n = 19)	146.11 ± 34.09 (n = 9)	153.96 ± 24.78 (n = 10)	0.608	0.467
24^+0^-27^+6^ weeks	160.43 ± 34.60 (n = 29)	148.55 ± 24.25 (n = 29)	146.66 ± 27.61 (n = 18)	161.32 ± 23.49 (n = 5)	143.60 ± 7.45 (n = 6)	0.136	0.345

APOA1, apolipoprotein A1; PAS, placenta accreta spectrum.

*p* ^*^, compared between the control and total PAS groups.

*p* ^**^, compared among the control group, accreta, increta and percreta groups.

a *p* < 0.05, compared with the control group.

b *p* < 0.05, compared with the placenta accreta groups.

c *p* < 0.05, compared with the placenta increta groups.

### 3.3 Association of plasma APOA1 levels with PAS-related outcomes

As is the case for all periods, the unadjusted analyses revealed a statistically significant association between maternal plasma APOA1 level and invasive PAS (OR = 0.980, 95% CI 0.970, 0.991, *p* < 0.001), PP (OR = 0.971, 95% CI 0.956, 0.987, *p* < 0.001), blood loss volume (β = -3.635, 95% CI -5.132, -2.138, *p* < 0.001), blood transfusion (OR = 0.982, 95% CI 0.971, 0.993, *p* = 0.002) and hysterectomy (OR = 0.974, 95% CI 0.958, 0.991, *p* = 0.003) (**Table 3**). After adjusting the model for maternal age, gravidity, parity, history of CS, history of curettage, placenta previa, and pre-pregnancy BMI, the association between plasma APOA1 level and invasive PAS, PP, blood loss volume, blood transfusion and hysterectomy remained statistically significant (*p* < 0.05). In subgroup analysis, such associations were still statistically significant during 32^+0^-35^+6^ weeks of gestation (*p* < 0.05). However, no statistically significant association was identified between maternal plasma APOA1 levels and PAS-related outcomes in 28^+0^-31^+6^ weeks and 24^+0^-27^+6^ weeks subgroups (*p* > 0.05).

**Table 3:** Associations of maternal plasma APOA1 level with PAS-related outcomes.

	Clinical outcome	β (95% CI)	OR (95% CI)	*p*
All periods (24^+0^-35^+6^ weeks)				
Crude				
	Invasive PAS	–	0.980 (0.970, 0.991)	< 0.001
	Placenta percreta	–	0.971 (0.956, 0.987)	< 0.001
	Blood loss volume (mL)	-3.635 (-5.132, -2.138)	–	< 0.001
	Vaginal delivery	-1.026 (-2.650, 0.598)	–	0.212
	CS	-4.417 (-6.444, -2.390)	–	< 0.001
	Blood transfusion	–	0.982 (0.971, 0.993)	0.002
	Vaginal delivery	–	0.975 (0.913, 1.042)	0.458
	CS	–	0.982 (0.970, 0.995)	0.006
	Hysterectomy	–	0.974 (0.958, 0.991)	0.003
Adjusted^*^				
	Invasive PAS	–	0.977 (0.961, 0.992)	0.003
	Placenta percreta	–	0.970 (0.950, 0.990)	0.003
	Blood loss volume (mL)	-2.528 (-3.868, -1.188)	–	< 0.001
	Vaginal delivery	-0.838 (-1.983, 0.306)	–	0.148
	CS	-3.499 (-5.283, -1.714)	–	< 0.001
	Blood transfusion^**^	–	0.981 (0.966, 0.997)	0.022
	CS	–	0.984 (0.968, 1.000)	0.047
	Hysterectomy	–	0.979 (0.959, 1.000)	0.048
32^+0^-35^+6^ weeks				
Crude				
	Invasive PAS	–	0.969 (0.952, 0.985)	< 0.001
	Placenta percreta	–	0.942 (0.914, 0.970)	< 0.001
	Blood loss volume (mL)	-4.637 (-7.096, -2.178)	–	< 0.001
	Vaginal delivery	-0.358 (-1.040, 1.756)	–	0.599
	CS	-7.532 (-12.124, -2.940)	–	0.002
	Blood transfusion^**^	–	0.971 (0.954, 0.988)	0.001
	CS	–	0.967 (0.948, 0.987)	0.001
	Hysterectomy	–	0.946 (0.916, 0.976)	0.001
Adjusted^*^				
	Invasive PAS	–	0.947 (0.916, 0.978)	0.001
	Placenta percreta	–	0.909 (0.856, 0.967)	0.002
	Blood loss volume (mL)	-3.756 (-5.926, -1.586)	–	0.001
	Vaginal delivery	-0.203 (-1.326, 0.920)	–	0.702
	CS	-6.116 (-8.907, -3.325)	–	< 0.001
	Blood transfusion^**^	–	0.937 (0.901, 0.975)	0.001
	CS	–	0.934 (0.892, 0.977)	0.003
	Hysterectomy	–	0.922 (0.866, 0.981)	0.010
28^+0^-31^+6^ weeks				
Crude				
	Invasive PAS	–	0.987 (0.969, 1.005)	0.146
	Placenta percreta	–	0.993 (0.972, 1.014)	0.520
	Blood loss volume (mL)	-2.616 (-6.767, 1.535)	–	0.213
	Vaginal delivery	-1.092 (-4.223, 2.038)	–	0.476
	CS	-2.620 (-8.392, 3.152)	–	0.367
	Blood transfusion^**^	–	1.014 (0.972, 1.057)	0.518
	CS	–	1.018 (0.975, 1.064)	0.419
	Hysterectomy	–	0.994 (0.970, 1.019)	0.638
Adjusted^*^				
	Invasive PAS	–	0.953 (0.855, 1.061)	0.378
	Placenta percreta	–	1.017 (0.979, 1.057)	0.390
	Blood loss volume (mL)	-0.521 (-4.693, 3.651)	–	0.804
	Vaginal delivery	-2.132 (-5.299, 1.035)	–	0.172
	CS	-0.839 (-5.619, 7.296)	–	0.795
	Blood transfusion^**^	–	1.014 (0.972, 1.057)	0.518
	CS	–	1.018 (0.975, 1.064)	0.419
	Hysterectomy	–	1.024 (0.980, 1.070)	0.291
24^+0^-27^+6^ weeks				
Crude				
	Invasive PAS	–	0.996 (0.974, 1.019)	0.727
	Placenta percreta	–	0.985 (0.953, 1.017)	0.351
	Blood loss volume (mL)	-1.419 (-6.004, 3.165)	–	0.538
	Vaginal delivery	-2.175 (-9.468, 5.119)	–	0.540
	CS	-0.102 (-6.173, 6.378)	–	0.974
	Blood transfusion	–	0.994 (0.971, 1.017)	0.586
	Vaginal delivery	–	0.975 (0.902, 1.053)	0.975
	CS	–	0.998 (0.970, 1.027)	0.878
	Hysterectomy	–	0.993 (0.961, 1.026)	0.667
Adjusted^*^				
	Invasive PAS	–	0.994 (0.946, 1.044)	0.805
	Placenta percreta	–	0.973 (0.883, 1.073)	0.584
	Blood loss volume (mL)	1.128 (-3.100, 5.356)	–	0.981
	Vaginal delivery	0.035 (-3.082, 3.152)	–	0.585
	CS	0.534 (-5.435, -6.503)	–	0.856
	Blood transfusion^**^	–	0.997 (0.945, 1.052)	0.925
	CS	–	0.992 (0.922, 1.068)	0.835
	Hysterectomy	–	0.985 (0.915, 1.061)	0.687

^*^Adjusted for maternal age, gravidity, parity, cesarean history, curettage history, placenta previa, pre-pregnancy BMI.

^**^The number of blood transfusions in the vaginal delivery group was minimal or null, the distribution of target variable categories was markedly imbalanced, and the logistic regression results were based solely on the constants in predicting the outcome of blood transfusion.

APOA1, apolipoprotein A1; PAS, placenta accreta spectrum; OR, odds ratio; CI, confidence interval; CS, cesarean section.

### 3.4 Predictive value of maternal plasma APOA1 level for PAS-related outcomes

**Figure 3:**
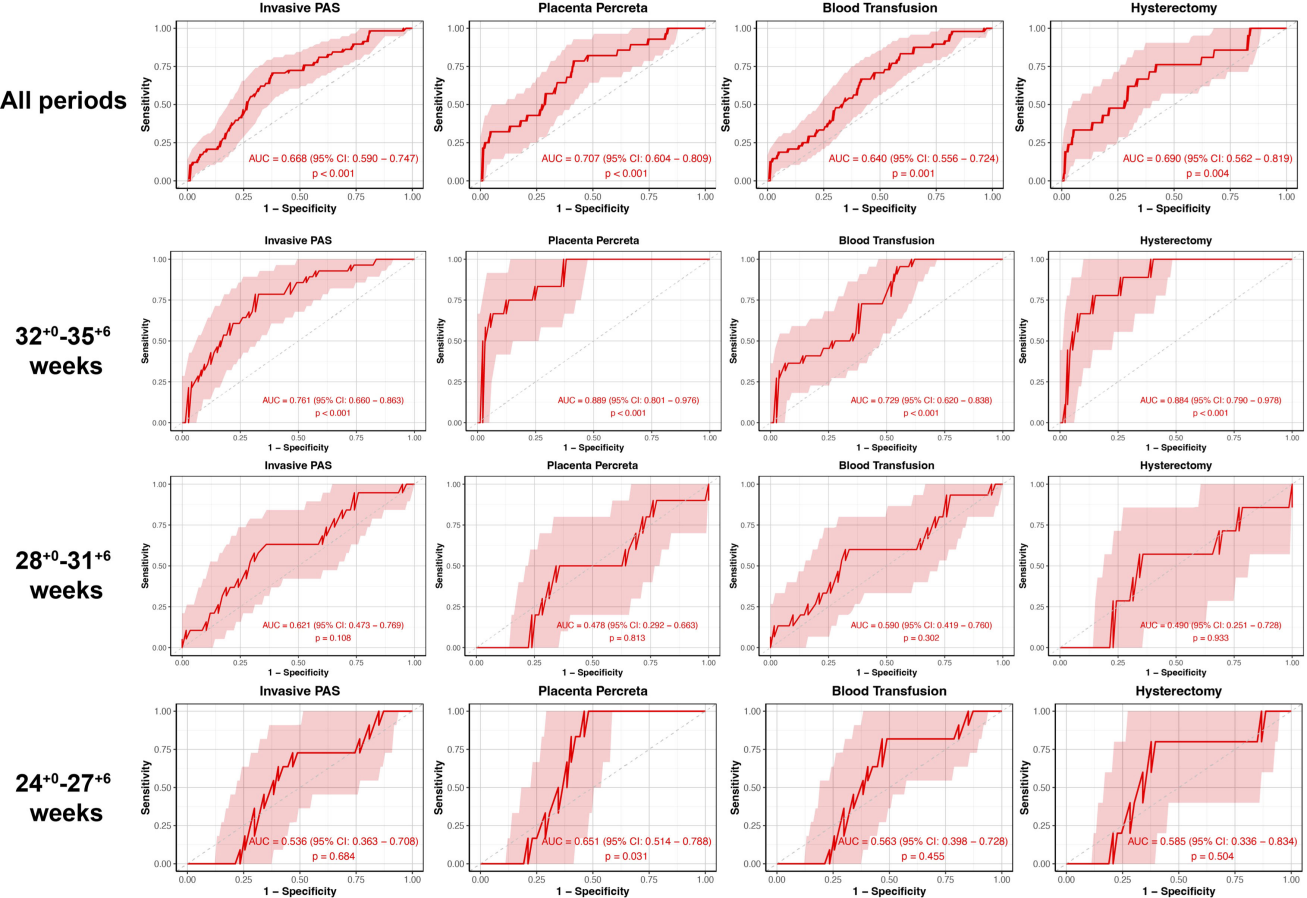
The ROC curve stratified by gestational age of plasma APOA1 levels to predict PAS-related outcomes. ROC, receiver-operating-characteristics; APOA1, apolipoprotein A1; PAS, placenta accreta spectrum.

The calculated AUC for the APOA1 levels in distinguishing invasive PAS, PP, blood transfusion and hysterectomy were 0.668 (95% CI 0.590-0.747, *p* < 0.001), 0.707 (95% CI 0.604-0.809, *p* < 0.001), 0.640 (95% CI 0.556-0.724, *p* = 0.001) and 0.690 (95% CI 0.562-0.819, *p* = 0.004), respectively, throughout all periods (**Figure 3**). In the subgroup of 32^+0^-35^+6^ weeks of gestation, the AUC for distinguishing invasive PAS, PP, blood transfusion and hysterectomy were 0.761 (95% CI 0.660-0.863, *p* < 0.001), 0.889 (95% CI 0.801-0.976, *p* < 0.001), 0.729 (95% CI 0.620-0.838, *p* < 0.001) and 0.884 (95% CI 0.790-0.978, *p* < 0.001), respectively. In the subgroup of 24^+0^-27^+6^ weeks of gestation, the AUC for distinguishing PP was 0.651 (95% CI 0.514-0.788, *p* = 0.031). No statistically significant difference was observed in the remaining outcomes (*p* > 0.05).

## 4 Discussion

### 4.1 Main findings

To our knowledge, this is the first study linking plasma APOA1 to PAS and its adverse outcomes. The present study revealed that the plasma APOA1 level of PAS patients was lower than that of the control group. During the gestational age between 24^+0^ and 35^+6^ weeks, the APOA1 levels of invasive PAS patients exhibited a gradual decline, with a more pronounced reduction observed in the PP group. A strong relationship was observed between APOA1 levels and invasive PAS, PP, blood loss volume, blood transfusion and hysterectomy, after adjusting for potential confounders during the period from 32^+0^ to 35^+6^ weeks of gestation. Plasma APOA1 may serve as a predictive marker for the occurrence of invasive PAS, PP, blood transfusion and hysterectomy.

### 4.2 Interpretation

The hallmark of PAS is the excessive invasion of placenta villi. Our findings indicated that a reduction in plasma APOA1 levels was associated with an increased severity of PAS. In contrast, preeclampsia is distinguished by shallow placental invasion and appears to exhibit an opposite pathogenesis compared to that of PAS (18). The plasma APOA1 level was observed to be significantly elevated in preeclampsia patients (16). The observation that APOA1 exhibits opposing changes in these two diseases with seemingly disparate etiologies serves to reinforce the hypothesis that APOA1 is inversely correlated with placental invasion. APOA1 is primarily produced and secreted by liver cells, although the placenta is also capable of releasing substantial quantities of it during pregnancy. An *in vitro* study demonstrated that trophoblast cells are capable of secreting a greater quantity of APOA1 than hepatocytes (21). It is traditionally been assumed that extravillous trophoblast cells (EVTs) exhibit tumor-like characteristics in PAS, including an enhanced capacity for proliferation, invasion and migration (22). Liu et al. demonstrated that APOA1 could inhibit the proliferation and invasion of trophoblast cells (16). This may provide an explanation for the observed reduction in APOA1 levels.

New evidence indicates that hypoxia, angiogenesis stimulation, and immune response suppression also contribute to the pathogenesis of PAS (23, 24). APOA1 has been demonstrated to facilitate vasorelaxation by stimulating endothelial nitric oxide production (25). The downregulation of APOA1 may be associated with vasocontraction and insufficient blood flow to the placenta, which in turn may result in angiogenesis to counteract hypoxia. Therefore, it can be surmised that a rich network of vessels will develop in the placenta and that a greater quantity of blood will be lost when patients with PAS delivery. Although there has been minimal research conducted on the immunological function of APOA1 during gestation, discoveries gained from gynecological tumor studies may provide some insights into this area. A significant decrease in plasma APOA1 level was observed in endometrium and ovarian cancers (26). A negative correlation was observed between the infiltration of CD163^+^ macrophages and APOA1, whereas a positive correlation was noted between the infiltration of CD8^+^ T cells and it (26). A comparable activation of M2 macrophages and inhibition of T cells were observed in PAS tissues (23). This suggests that APOA1 may play a role in the pathogenesis of PAS by modulating the function of macrophages and T cells.

The study conducted by Dathan-Stumpf et al. (27) indicated that there was no discernible alteration in APOA1 levels between the second and third trimesters. This finding aligns with the results observed in the non-PAS group in our study. However, our study provides a more detailed account of APOA1 changes after 24 weeks of gestation, thereby supplementing the existing data on PAS patients. The different characteristics of APOA1 alterations may be associated with the activity of placental invasion in the late trimesters. The status of epithelial-to-mesenchymal transition (EMT) was observed to remain active in the third trimester of pregnancy, indicating the continued differentiation of cytotrophoblasts (CTBs) into EVTs (28). Transcription factors associated with EMT were observed to be overexpressed in the increta region of the placenta (23). In lung fibrosis, APOA1 has been demonstrated to inhibit the EMT process (29), which suggests that a low level of APOA1 may stimulate EMT and promote the differentiation of CTB-EVT, thereby maintaining the invasion capacity of placental villi in the third trimester. This may elucidate the mechanism that underlies the observed correlation between APOA1 level and the severity of PAS.

### 4.3 Strengths and limitations

This is the first study to examine the correlation between maternal plasma APOA1 levels and PAS, as well as its severity and adverse outcomes. Previous studies have explored various blood biomarkers for PAS, including aneuploidy screening markers, as well as parameters associated with angiogenesis, oxidative stress, coagulation, and immune responses (12, 30). However, these investigations were typically limited by small sample sizes in the PAS groups, with most studies including fewer than 50 cases and rarely exceeding 100 cases. In contrast, our study retained 118 PAS cases after PSM, representing one of the largest sample sizes among published PAS biomarker studies to date, while additionally controlling for potential confounding factors. Moreover, due to the low incidence of PAS and consequent sample size limitations, previous studies generally failed to stratify PAS severity. Our study successfully conducted subgroup analyses across PA, PI and PP with statistically significant results. Finally, APOA1 is a practical and widely available biomarker, measurable in standard hospital labs. While MRI remains valuable for diagnosing invasive PAS, its accuracy varies by operator experience and accessibility is limited in many settings. In contrast, APOA1 testing is cost-effective, highly standardized, and available in most hospitals. These advantages make APOA1 a promising screening tool, particularly where MRI access is restricted. With further validation in our ongoing multicenter study, APOA1 could be integrated into clinical practice within short time.

Some limitation of this study should be listed. The present study did not examine maternal APOA1 levels in the first trimester of gestation. The early evaluation of APOA1 may facilitate the prediction process. Secondly, there was no accurate uniformity in the time of APOA1 level assessment. This is attributable to the fact that peripheral blood was collected when necessary test, such as oral glucose tolerance test, were conducted in accordance with standard clinical practice, with minimal additional burden to the pregnant women. Ultimately, the underlying mechanism by which APOA1 exerts its influence on PAS remains unclear.

## 5 Conclusion

The current study demonstrates a reduction in plasma levels of APOA1 in patients with PAS, particularly within the PP group. The trajectory of APOA1 levels exhibited a decline from 24^+0^ to 35^+6^ weeks of gestation in patients with invasive PAS. Furthermore, a decreased level of APOA1 was found to be associated with the severity of PAS and its adverse outcomes, including blood transfusion and hysterectomy, under multidisciplinary team management and standardized surgical protocols.

## Data Availability

The raw data supporting the conclusions of this article will be made available by the authors, without undue reservation.
